# Evaluating methods for the analysis of rare variants in sequence data

**DOI:** 10.1186/1753-6561-5-S9-S119

**Published:** 2011-11-29

**Authors:** Alexander Luedtke, Scott Powers, Ashley Petersen, Alexandra Sitarik, Airat Bekmetjev, Nathan L Tintle

**Affiliations:** 1 Division of Applied Mathematics, Brown University, 182 George Street, Providence, RI 02912, USA; 2 Department of Statistics and Operations Research, 318 Hanes Hall, CB 3260, University of North Carolina, Chapel Hill, NC 27599-3260, USA; 3 Departments of Mathematics, Computer Science, and Statistics, St. Olaf College, 1520 St. Olaf Avenue, Northfield, MN 55057, USA; 4 Department of Mathematics, Wittenberg University, 200 West Ward Street, PO Box 720, Springfield, OH 45501, USA; 5 Department of Mathematics, Computer Science and Statistics, Dordt College, 498 4th Ave NE, Sioux Center, IA 51250, USA

## Abstract

A number of rare variant statistical methods have been proposed for analysis of the impending wave of next-generation sequencing data. To date, there are few direct comparisons of these methods on real sequence data. Furthermore, there is a strong need for practical advice on the proper analytic strategies for rare variant analysis. We compare four recently proposed rare variant methods (combined multivariate and collapsing, weighted sum, proportion regression, and cumulative minor allele test) on simulated phenotype and next-generation sequencing data as part of Genetic Analysis Workshop 17. Overall, we find that all analyzed methods have serious practical limitations on identifying causal genes. Specifically, no method has more than a 5% true discovery rate (percentage of truly causal genes among all those identified as significantly associated with the phenotype). Further exploration shows that all methods suffer from inflated false-positive error rates (chance that a noncausal gene will be identified as associated with the phenotype) because of population stratification and gametic phase disequilibrium between noncausal SNPs and causal SNPs. Furthermore, observed true-positive rates (chance that a truly causal gene will be identified as significantly associated with the phenotype) for each of the four methods was very low (<19%). The combination of larger than anticipated false-positive rates, low true-positive rates, and only about 1% of all genes being causal yields poor discriminatory ability for all four methods. Gametic phase disequilibrium and population stratification are important areas for further research in the analysis of rare variant data.

## Background

Genome-wide association studies (GWAS) hope to identify variations in the human genome that increase disease risk. Over the past decade, single-nucleotide polymorphism (SNP) microarrays have been used in GWAS to explore the association of common variants with disease. With the advent of next-generation sequencing technology, consideration of rare variants is now possible. A number of rare variant methods [1-4] have been recently proposed as first attempts to investigate the contribution of rare genetic variants to common disease. These methods all take a similar approach in which variants (SNPs) are aggregated at the gene level. Specifically, all variants within a gene are assigned to that gene, and the methods are designed to test whether, in total, the variants in the gene show association with the phenotype. To date, there has been no systematic comparison of the proposed methods. Furthermore, there has been little to no application of these methods to actual sequence data, and so little is known about the practical issues that will arise when applying these methods to real data.

In this paper, we use real genotypes and simulated phenotype data from Genetic Analysis Workshop 17 (GAW17) to provide a systematic and comprehensive comparison of the power and type I error of each of four rare variant methods (combined multivariate and collapsing, weighted sum, proportion regression, and cumulative minor allele test) in a variety of scenarios. This comparison gives practical insights into power and sample size issues in the analysis of next-generation sequencing data in the new wave of GWAS and suggests further areas of research needed to improve type I error and power in practice.

## Methods

### Data

All analyses presented here are based on data provided by the organizers of GAW17. Detailed descriptions of the data and simulation of the disease phenotype are provided elsewhere [5]. We provide a brief overview here. The data consist of 697 unrelated individuals genotyped at 24,487 autosomal SNPs contained in at least 1 of 3,205 different genes. We consider three sets of SNPs. The first set is all 21,355 SNPs with minor allele frequency (MAF) < 0.05; the second set is a superset of the first, containing all 24,487 autosomal SNPs; and the last set is a subset of the second set, containing the 13,572 SNPs that are bioinformatically predicted to be nonsynonymous. Because some genes contain only SNPs with MAF > 5% or only synonymous SNPs, the total number of genes under analysis is reduced when analyzing these subsamples (the analysis for SNPs with MAF < 5% uses 2,874 total genes; the analysis for synonymous SNPs uses 2,196 total genes). All SNP genotypes are coded as 0 or 1, where 0 means no copies of the minor allele are present and 1 means that at least one copy of the minor allele is present. This coding strategy for SNP genotypes is required by some of the analytic methods considered and is a reasonable choice for rare variants. For common variants, of only minor importance in our analysis, the code represents the presumption of a dominant disease model.

The organizers of GAW17 simulated a dichotomous disease phenotype (Affected/Not affected) onto the 697 individuals, and that phenotype is the focus of our analyses in this paper. The dichotomous disease phenotype is caused by a combination of measured SNPs (162 SNPs in 37 genes) and unmeasured SNPs. Two-hundred separate simulated phenotype replicates (each based on the same disease model) were produced. We note that the focus of our analysis is SNPs with MAF < 5% because the methods being compared were designed for use on rare variants, although the other analyses (all SNPs and only nonsynonymous SNPs) are included for comparison.

### Rare variant methods

We compare four rare variant statistical methods: combined multivariate and collapsing, weighted sum, proportion regression, and cumulative minor allele test. A detailed discussion of the first three methods is provided by Dering et al. [6]. We provide brief overviews of these three methods here and a more detailed description of the fourth method.

The combined multivariate and collapsing (CMC) method [1] combines SNPs within a gene into subgroups based on some criterion (e.g., MAF threshold) and then applies a multivariate test (e.g., Hotelling *T*^2^) to the groups to obtain a test statistic. In our implementation of the CMC method we group all SNPs in a gene that have MAF < 0.01 into one subgroup, and all other SNPs belong to their own groups. We use the Hotelling *T*^2^ statistic and its asymptotic distribution to assess statistical significance.

The next method we consider is the weighted-sum (WS) method, which calculates a score for each individual by summing the ratio of the genotype to the estimated standard deviation of the variant (under the null hypothesis) across all variants within the gene [2], effectively putting more weight on variants with lower MAFs. Scores are then used to rank the individuals, and a test statistic is obtained by summing the rank of gene scores for the affected individuals. One thousand phenotype permutations are used to assess the significance of the WS statistic.

The third method we consider is the proportion regression (PR) method [3], in which the proportion of variant sites within a gene containing the rare variant is regressed against the phenotype. As proposed by Morris and Zeggini [3], we use the asymptotic test for logistic regression to evaluate significance.

The last method we consider is the cumulative minor allele test (CMAT), which uses a 2 × 2 chi-square statistic to compare the total numbers of rare variants present in the gene for case subjects and control subjects [4]. Specifically, the CMAT compares two proportions using a chi-square statistic: (1) the proportion of rare alleles in case subjects, computed as the total number of rare alleles present within the gene of interest totaled across the case group divided by the total number of SNP loci within the gene of interest multiplied by the number of case subjects; and (2) the proportion of rare alleles in the control subjects, computed as the total number of rare alleles present within the gene of interest totaled across the control group divided by the total number of SNP loci within the gene of interest multiplied by the number of control subjects. Because of the potential for linkage disequilibrium between markers and small counts, the significance of the CMAT statistic is determined by phenotype permutation (1,000 permutations) instead of the chi-square distribution.

### Gene and sample characteristics

In our analysis we use a variety of measures to help assess power and type I error patterns. We define a measure of population stratification (*P*) for each of the 200 replicates by finding the value of the Pearson chi-square test of association between the dichotomous phenotype and the ethnicity of each person in the sample (CEPH [European-descended residents of Utah], Luhya, Yoruba, Japanese, Denver Chinese, Han Chinese, and Tuscan).

We define a spuriously associated gene as any gene that is identified as significantly (*p* < 0.05) associated with the phenotype in at least 16 of the 200 replicates for all four methods when analyzing only SNPs with MAF < 5%, but that is not actually associated with the disease phenotype. We use a value of 16 because it is the 96th percentile of a binomial distribution (Bin(*n* = 200, *p* = 0.05)) representing the distribution of the number of *p*-values less than 0.05 under the assumption of independence of *p*-values within the gene and across replicates when the type I error rate is 5%. We note that the assumption of independence may not be valid because the genotypes are constant across all 200 replicates. However, the binomial distribution model serves as a reasonable starting point for considering inflated type I errors. In addition, our goal in identifying spuriously associated genes is to explore characteristics of these genes, and so we choose to use a sensitive criterion (0.05) instead of one that adjusts for multiple testing.

Gametic phase disequilibrium occurs when correlation between loci occurs beyond what would be expected by random chance [7]. We define a measure of gametic phase disequilibrium (*G*) as the aggregate amount of correlation between a noncausal SNP and the 162 causal SNPs. Specifically, *G* is the *r*^2^ from a regression model obtained by regressing the genotype of the noncausal SNP on the genotypes of the 162 causal SNPs.

We also define an overall measure of gametic phase disequilibrium for the entire sample. To do this, we use the matrix of genotypes of the 24,487 SNPs in the mini-exome scan for 697 people (each row is a person; each column represents a different SNP). We first choose 100,000 random pairs of columns, where each pair represents SNPs on different chromosomes (to ensure no true linkage disequilibrium between SNPs). We then compute an overall measure of SNP correlation *S* as the average of the squared correlations between the genotypes of all 24,487 SNPs across the 697 people:(1)

where  is the squared correlation between the *i*th randomly selected pair of SNPs.

## Results

In the data under analysis, 36 of 3,205 (1.12%) genes are associated with a simulated phenotype. Table 1 summarizes the results of the four analytic methods run on all genes across all 200 phenotype replicates. As noted earlier, the total number of genes under analysis varies depending on the sample being analyzed: 2,874 for only rare SNPs (MAF < 5%), 3,205 genes for all SNPs, and 2,196 for all nonsynonymous SNPs. At nominal significance levels of 5% and 0.5% in the analysis that was limited to rare variant SNPs, the PR method identified the highest percentage of true discoveries (2.41% and 4.20% of the genes identified as significant) followed by the CMAT, WS, and CMC methods. However, these values are only marginally higher than would be obtained by randomly choosing genes (1.12%).

**Table 1:** Overall ability of the four rare variant methods to identify genes as significantly associated with the phenotype

Method	Nominal *α* = 0.05			Nominal *α* = 0.005		
	
	Total number of significant associations	Number of significant associations that are actually causal	True discoveries (%)	Total number of significant associations	Number of significant associations that are actually causal	True discoveries (%)
Only SNPs with MAF < 5%						
WS	281.0	5.69	2.03	52.5	1.56	2.97
CMAT	201.4	4.42	2.19	38.4	1.31	3.44
CMC	256.5	3.80	1.48	38.2	0.92	2.41
PR	184.6	4.46	2.41	27.9	1.17	4.20
All SNPs						
WS	348.7	6.81	1.95	76.1	2.05	2.69
CMAT	294.9	4.63	1.57	63.8	1.46	2.28
CMC	361.1	5.16	1.43	64.6	1.23	1.90
PR	285.6	4.74	1.66	53.7	1.39	2.59
Nonsynonymous SNPs only						
WS	206.1	5.25	2.54	42.3	1.78	4.20
CMAT	173.3	4.19	2.42	35.7	1.44	4.03
CMC	223.3	4.99	2.23	38.5	1.33	3.46
PR	168.7	3.88	2.30	29.4	1.38	4.69

All values are averaged over 200 replicates. WS, weighted sum; CMAT, cumulative minor allele test; CMC, combined multivariate and collapsing; PR, proportion regression.

Table 1 also illustrates how these values are affected when using all SNPs (including those with MAF > 5%) and when considering only nonsynonymous SNPs. In both cases, these changes have only modest effect on the true-positive rate such that no method yielded a true-positive rate greater than 5% in any scenario.

### False-positive rate

As a first step in exploring why the true discovery rate is so low for all four methods, we compared the false-positive rate (nominal *α* = 0.05) across the genes that do not contain a causal SNP. The false-positive rate is computed as the proportion of times a gene-replicate combination is found to have a *p*-value less than 0.05 out of all total gene-replicate combinations for each of the noncausal genes. Overall false-positive rates for each of the four methods are found in Table 2.

**Table 2:** Overall false- and true-positive rates for the four rare variant methods (significance level 5%)

Rare variant method	False-positive rate (%)			True-positive rate (%)		
	
	Only SNPs with MAF < 5%	All SNPs	Only nonsynonymous SNPs	Only SNPs with MAF < 5%	All SNPs	Only nonsynonymous SNPs
WS	9.7	10.8	9.3	15.8	18.9	14.6
CMAT	6.9	9.2	7.8	12.3	12.8	11.6
CMC	8.9	11.2	10.1	10.6	14.3	13.9
PR	6.3	8.9	7.6	12.4	13.2	10.8

All values are averaged over 200 replicates. WS, weighted sum; CMAT, cumulative minor allele test; CMC, combined multivariate and collapsing; PR, proportion regression.

Although we would expect the false-positive rate to be at or near the nominal significance level of 5%, we see increased rates across all methods. These increased rates can be pinpointed to a subset of the genes that showed consistent association with the phenotype across replicates and across the four analytic methods. When analyzing rare variants only, we identified 561 spuriously associated genes out of 2,838 truly noncausal genes (see Methods section for details), leaving 2,277 noncausal genes that are not classified as spuriously associated with the phenotype.

We then performed further analyses to better understand the characteristics of the spuriously associated genes. We found that, in general, spuriously associated genes contained significantly more SNPs in the gene (average of 13.9 vs. 6.9, *p* < 2.2 × 10^−16^) and significantly higher average values of *G* among the SNPs in the gene (average of 3.47 vs. 1.87, *p* = 6.4 × 10^−14^). We fitted a multiple logistic regression model to predict whether or not a gene was spuriously associated with the number of SNPs in the gene, using the number of SNPs in the gene, the average MAF of the SNPs in the gene, the average value of *G* of SNPs in the gene, and all possible interactions between these variables. We found a significant interaction between the number of SNPs in the gene and *G*. The multiple regression model implicated a combination of a large number of SNPs and larger values of *G* (larger gametic phase disequilibrium with causal SNPs) as having synergistic effects in increasing the likelihood of a gene being identified as spuriously associated with the phenotype.

In addition to gene characteristics as the cause of the increased type I error rate, another possible reason for the inflated type I errors is population stratification. Using the measure of population stratification *P* computed for each of the 200 replicates, we computed the Pearson correlation between population stratification and the number of genes found as significant by each method in each of the 200 replicates. All four methods showed significant positive correlation (*p* < 6.5 × 10^−4^ in all cases) between the measure of population stratification and the number of significantly associated genes in the replicate (Pearson correlations: 0.24 for the CMC method, 0.28 for the PR method, 0.26 for CMAT, and 0.26 for the WS method).

To assess the overall effect of both gene characteristics and sample characteristics on type I error rate, we used multiple logistic regression. A data set was created for each of the four methods, with one row for each gene-replicate combination. We created a logistic regression model to predict whether or not the gene-replicate combination was significantly (*p* < 0.05) associated with the phenotype using gene variables (number of SNPs and *G*) as well as *P* (sample variable). All four logistic regression models (one for each of the four rare variant methods), showed significant association for all three variables, suggesting that all factors are contributing to the inflated type I error rate present in the analysis.

### True-positive rate

The next step in comparing the four different rare variant methods was to compute the power of each method across the 36 genes containing causal SNPs at a nominal type I error rate of 0.05. The overall power was computed as the number of times a gene-replicate combination was found to have a *p*-value less than 0.05 out of 36 × 200 total gene-replicate combinations. Overall power values for each of the four methods are given in Table 2.

As expected, all methods showed a strong correlation between the number of times each gene was found to be significant (*p* < 0.05, out of 200 times) and a measure of each gene’s association with the phenotype (MAF times *β* [risk] summed across all SNPs in the gene). Pearson correlations were 0.63 (CMAT), 0.63 (CMC method), 0.64 (PR method) and 0.54 (WS method) across the 36 causal genes.

The WS method showed significantly higher overall power (18.9%) compared to the other three methods, which were all comparable. Regression models suggest that the main reason for the apparent power increase for the WS method may be the number of noncausal SNPs in the gene (the more noncausal SNPs, the more likely the WS method was to find the gene statistically significant) (details not shown).

### Other analyses

We separately analyzed only nonsynonymous SNPs and the full set of all SNPs (regardless of function and MAF). Our analyses showed that restricting the set of SNPs to only nonsynonymous SNPs resulted in a minor improvement in the true-positive rate compared to analyzing the entire set of rare variants but that analyzing all SNPs resulted in a decreased true-positive rate (see Table 1). In addition, larger false-positive rates and lower true-positive rates were observed when all SNPs were analyzed simultaneously, whereas false-positive rates were only modestly increased when using only nonsynonymous SNPs, with a comparable true-positive rate (see Table 1).

We also found evidence of gametic phase disequilibrium between SNPs beyond what we would expect to happen purely randomly. We computed *S* for the genotype matrix and observed a value of 0.00241. We then independently permuted the rows within each column of the SNP genotype matrix and recomputed the value of *S* 1,000 times. The maximum value obtained through permutation was 0.0017. Because *S* has an approximately normal distribution under permutation, we estimated the mean and standard deviation and calculated a *Z* score for the observed data as 8.6, a large value significantly beyond what would be expected to happen by random chance.

### Considering population stratification

To more formally assess the effect of population stratification on the analysis, we used the covariate adjustment procedure outlined for CMAT [4], stratifying the sample on each of the seven subpopulations present in the sample. We reran the stratified CMAT procedure on all 200 replicates for each of the 2,874 genes containing a SNP with MAF < 5%. The analysis showed some improvement over the non-population-stratified analysis, although the results were still far from optimal. Specifically, the stratified CMAT yielded an average of 164.4 significant associations in each replicate for a nominal *α* of 0.05, lower than the other four methods (compare to Table 1); however, the percentage of true discoveries was second worst (1.89%; 3.11 true discoveries per replicate on average). Similar results were found when *α* was set to 0.005. The false-positive rate was the lowest of all methods (5.7%; compare to Table 2); however, the true-positive rate was also the lowest (8.6%). Most important, in a multiple logistic regression analysis assessing the overall effect of gene and sample characteristics on the type I error rate (corresponding analysis in the last paragraph of the “False-Positive Rate” subsection), all three gene characteristic variables were significant (*p* < 0.05).

## Discussion

Application of recently proposed methods for analyzing rare variant data has identified a number of important considerations for their use in practice. Specifically, all methods suffered from increased false-positive and decreased true-positive rates.

Paramount among the problems of the rare variant methods was an inflated false-positive rate. Our analyses suggest a number of gene-specific characteristics and sample characteristics that contribute to the increased false-positive rate. First, all methods showed increased false-positive rates for genes containing more SNPs. These results were further confirmed by analysis showing better false-positive control when fewer SNPs were used in the analysis (e.g., SNPs with MAF < 5% or only nonsynonymous SNPs). That all methods suffered from increased false positives demonstrates the need for improved rare variant methods that are less prone to type I errors in these cases.

Second, gametic phase disequilibrium was strongly associated with type I error. A typical assumption in simulations presented for method development is that little to no linkage disequilibrium is present in the analysis of rare variant data because of the low allele frequencies. This may be the case at the *population* level; that is, the data are unlikely to exhibit linkage disequilibrium between rare variants with the same gene in any population of interest. However, in any practical analysis of sequence data, for which the number of individuals genotyped is significantly less than the number of variant sites genotyped, it is entirely plausible that two rare variants *even on different chromosomes* may realize their minor allele within the same individual. Or at least, two rare variants may seem to be correlated merely because each of them occurs only in a few individuals, and some of those individuals are, by chance, the same for both variants. As shown (in the “Other Analyses” subsection of the Results section), some gametic phase disequilibrium is expected because of the finite sample size, but the gametic phase disequilibrium observed in the sample is higher than would have occurred by random chance alone, suggesting systematic genotyping errors.

Third, population stratification was identified as a potential cause of the increased false positives (see the “False-Positive Rate” subsection of the Results section). This result was confirmed when an additional analysis using the stratified CMAT showed improved type I error rates. The investigators proposing the other three methods (PR, CMC, and WS) give limited attention to population stratification. Further work is necessary to explore approaches for handling population stratification in rare variant tests.

All methods also suffered from low true-positive rates. The 36 causal genes analyzed here represent a range of number of causal SNPs, MAFs, and risks. Expectedly, the more causal SNPs in the gene, the larger their MAFs; and the larger the risk, the more power. However, if the 36 genes in this study are well representative of the true distribution of causal SNPs, MAFs, and risks in practice, then there is some need for concern over the low power to detect these associations.

## Conclusions

Recently proposed rare variant methods showed limited ability to identify causal genes in the analysis of mini-exome scan data because of decreased true-positive and increased false-positive error rates. Increased error rates were due to the presence of gametic phase disequilibrium and population stratification. In general, the methods also showed oversensitivity to the inclusion of noncausal variants, suggesting that using only nonsynonymous SNPs and SNPs with MAF < 5% can yield modestly improved results.

## Competing interests

The author(s) declare that there is/are no competing interest(s).

## Authors’ contributions

NT and AB designed the study and directed the research. AL, SP, AP and AS implemented the study and analyzed results. AL, SP and NUT drafted the manuscript. All authors read and approved the final manuscript.

## References

[B1] Madsen BE Browning SR A groupwise association test for rare mutations using a weighted sum statistic PLoS Genet 2009 5 e1000384 10.1371/journal.pgen.1000384 19214210 PMC2633048

[B2] Li B Leal SM Methods for detecting associations with rare variants for common diseases: application to analysis of sequence data Am J Hum Genet 2008 83 311 321 10.1016/j.ajhg.2008.06.024 18691683 PMC2842185

[B3] Morris A Zeggini E An evaluation of statistical approaches to rare variant analysis in genetic association studies Genet Epidemiol 2010 34 188 193 10.1002/gepi.20450 19810025 PMC2962811

[B4] Zawistowski M Gopalakrishnan S Ding J Li Y Grimm S Zollner S Extending rare variant testing strategies: analysis of non-coding sequence and imputed genotypes Am J Hum Genet 2010 87 604 617 10.1016/j.ajhg.2010.10.012 21070896 PMC2978957

[B5] Almasy LA Dyer TD Peralta JM Kent JW Jr. Charlesworth JC Curran JE Blangero J Genetic Analysis Workshop 17 mini-exome simulation BMC Proc 2011 5 suppl 9 S2 22373155 10.1186/1753-6561-5-S9-S2 PMC3287854

[B6] Dering C Pugh E Ziegler A Statistical analysis of rare sequence variants: an overview of collapsing methods Genet Epidemiol 2011 3 Suppl 8 12 17 10.1002/gepi.20643 PMC3277891 22128052

[B7] Lewontin RC On measures of gametic disequilibrium Genetics 1988 120 849 852 3224810 10.1093/genetics/120.3.849 PMC1203562

